# Integrative microRNA and transcriptome analysis reveals sex-specific molecular divergence in human bladder cancer

**DOI:** 10.1186/s13293-026-00829-5

**Published:** 2026-01-23

**Authors:** Yizhou Wang, Priyanka Bhandary, Jason H. Moore, Xue Li, Zhiping Wang

**Affiliations:** 1 Department of Computational Biomedicine, Cedars Sinai Medical Center, Los Angeles, CA USA; 2 Department of Medicine and Department of Biomedical Sciences, Cedars Sinai Medical Center, Los Angeles, CA USA; 3 Samuel Oschin Comprehensive Cancer Institute, Cedars Sinai Medical Center, Los Angeles, CA USA

## Abstract

**Background:**

Bladder cancer affects men and women differently: men are diagnosed more frequently, but women often present with advanced disease and have worse survival. The biological mechanisms underlying these disparities remain unclear. This study aimed to identify sex-specific molecular features and regulatory interactions that shape tumor biology and outcomes.

**Methods:**

We performed an integrative multi-omics analysis combining bulk messenger RNA and microRNA expression, survival modeling, and single-cell transcriptomic profiling. Data were obtained from The Cancer Genome Atlas, Gene Expression Omnibus, and the Genome Sequence Archive. Differential expression analyses were conducted separately in tumors and in normal samples to compare males and females. Experimentally validated microRNA–mRNA target pairs were tested for correlation, and survival associations were evaluated using Kaplan–Meier and Cox models. Single-cell RNA-seq data were analyzed to assess sex-biased expression across tumor and immune cell populations.

**Results:**

We identified 48 tumor-specific sex-biased microRNAs and 456 tumor-specific sex-biased genes, the majority located on autosomes rather than sex chromosomes. Correlation analysis revealed 82 experimentally supported, negatively correlated microRNA–mRNA pairs, including 63 discordant pairs with opposite sex-biased expression, suggesting sex-specific regulatory interactions. Several of these features were significantly associated with overall survival in a sex-dependent manner. For example, the male-upregulated microRNA miR-1270 showed repression of the female-biased targets CYP26B1 and FAM180A, both of which were associated with poor survival, highlighting potential prognostic and therapeutic relevance. Single-cell analysis revealed widespread sex-biased expression across epithelial, stromal, and immune cells, with female tumors showing stronger signals in stromal and immune compartments, which may contribute to the more aggressive clinical course observed in females.

**Conclusions:**

Our findings indicate that sex disparities in bladder cancer are largely driven by post-transcriptional regulation of autosomal genes, rather than sex chromosome dosage. By linking sex-biased microRNAs, target genes, and patient survival with cell type–specific expression, this study provides new insight into the biological basis of sex differences in bladder cancer. These results underscore the importance of incorporating sex as a critical variable in biomarker development, therapeutic targeting, and clinical trial design.

**Supplementary Information:**

The online version contains supplementary material available at 10.1186/s13293-026-00829-5.

## Abstract (plain language summary)

Bladder cancer affects men and women differently. Men are more likely to develop the disease, but women who are diagnosed often have more advanced cancer and worse survival. The reasons for these differences are not fully understood.

In this study, we looked for biological explanations for these sex differences. Using data from large cancer research programs, we examined the activity of thousands of genes and small molecules called microRNAs (miRNAs), which help control gene activity. We also analyzed single cells from bladder tumors to see which genes were active in different cell types.

We found 48 miRNAs and 456 genes that differ between male and female bladder tumors. Most of these differences were not on sex chromosomes, but on other chromosomes, showing that regulation of gene activity plays a major role. Some of these miRNAs and genes were also linked to survival, but only in men or only in women.

At the single-cell level, we found that sex-biased genes were especially active in immune cells and stromal cells that support tumor growth. This pattern may help explain why female bladder tumors can behave more aggressively.

Overall, our results show that sex is an important factor in bladder cancer biology. Taking these differences into account could improve diagnosis, guide treatment choices, and ensure that future clinical trials benefit both men and women.

**Supplementary Information:**

The online version contains supplementary material available at 10.1186/s13293-026-00829-5.

## Highlights

We performed an integrative analysis of bulk mRNA, miRNA, survival data, and single-cell RNA-seq to investigate sex differences in human bladder cancer.

A total of 48 miRNAs and 456 genes showed sex-biased activity in tumors, the vast majority located on autosomes rather than sex chromosomes.

We identified 82 validated miRNA–mRNA pairs, including discordant pairs in which male-upregulated miRNAs target female-upregulated genes (or vice versa), consistent with post-transcriptional repression that differs by sex.

Several sex-biased miRNAs and genes showed sex-stratified associations with overall survival, suggesting potential prognostic biomarkers.

Single-cell analysis revealed that sex-biased genes were especially active in immune and stromal cells, providing insight into why female bladder tumors often present as more aggressive.

**Supplementary Information:**

The online version contains supplementary material available at 10.1186/s13293-026-00829-5.

## Introduction

Cancer exhibits well-established differences in both incidence and survival outcomes between males and females [1, 2], with underlying causal factors extending beyond the contributions of sex chromosomes and systemic hormones [3–5]. Bladder cancer, in particular, displays a striking sex disparity: while men are diagnosed at significantly higher rates [6–8], women more frequently present with advanced-stage disease and experience poorer overall survival [9]. Although behavioral and environmental exposures such as tobacco use and occupational contact with carcinogens partially explain the higher incidence in men, accumulating evidence points to biological mechanisms as central contributors to these sex-based differences [10].

Recent studies suggest that these disparities are not solely attributable to chromosomal or hormonal differences [11–13]. Instead, sex-specific variations in tumor biology including differential gene expression, immune responses, and epigenetic regulation have been identified. For instance, female bladder tumors often exhibit elevated expression of immune checkpoint molecules and a more immunosuppressive tumor microenvironment, potentially contributing to their worse clinical outcomes [14]. Moreover, sex-based differences in urothelial cell biology, DNA damage repair pathways, and hormone-mediated effects on the local bladder environment have also been reported [15, 16]. These findings highlight the need for integrative, mechanistic studies encompassing genomics, immunology, and endocrinology to unravel the complex biological basis of sex disparities in bladder cancer and to guide the development of sex-informed prevention and treatment strategies.

Our previous study [17] investigated sex-specific genetic biomarkers in bladder cancer and identified both previously reported genes, such as *DLGAP5*, *SOX2*, *LAMA2*, and *COL5A2*, as well as novel candidates including *ERCC5*, *NID1*, and *ANK2*. These findings support the existence of sex-associated molecular differences in bladder cancer. However, the underlying regulatory mechanisms that drive these sex-specific gene expression patterns remain poorly understood. In the present study, we focus on microRNAs (miRNAs), which have been widely recognized as key post-transcriptional regulators of gene expression in cancer [18–23]. miRNAs can function as oncogenes or tumor suppressors, influencing critical cellular processes such as proliferation, apoptosis, invasion, metastasis, and therapeutic resistance. Despite extensive research on the role of miRNAs in various cancers, their contribution to sex-based differences in bladder cancer has not, to our knowledge, been systematically examined.

**Fig. 1:**
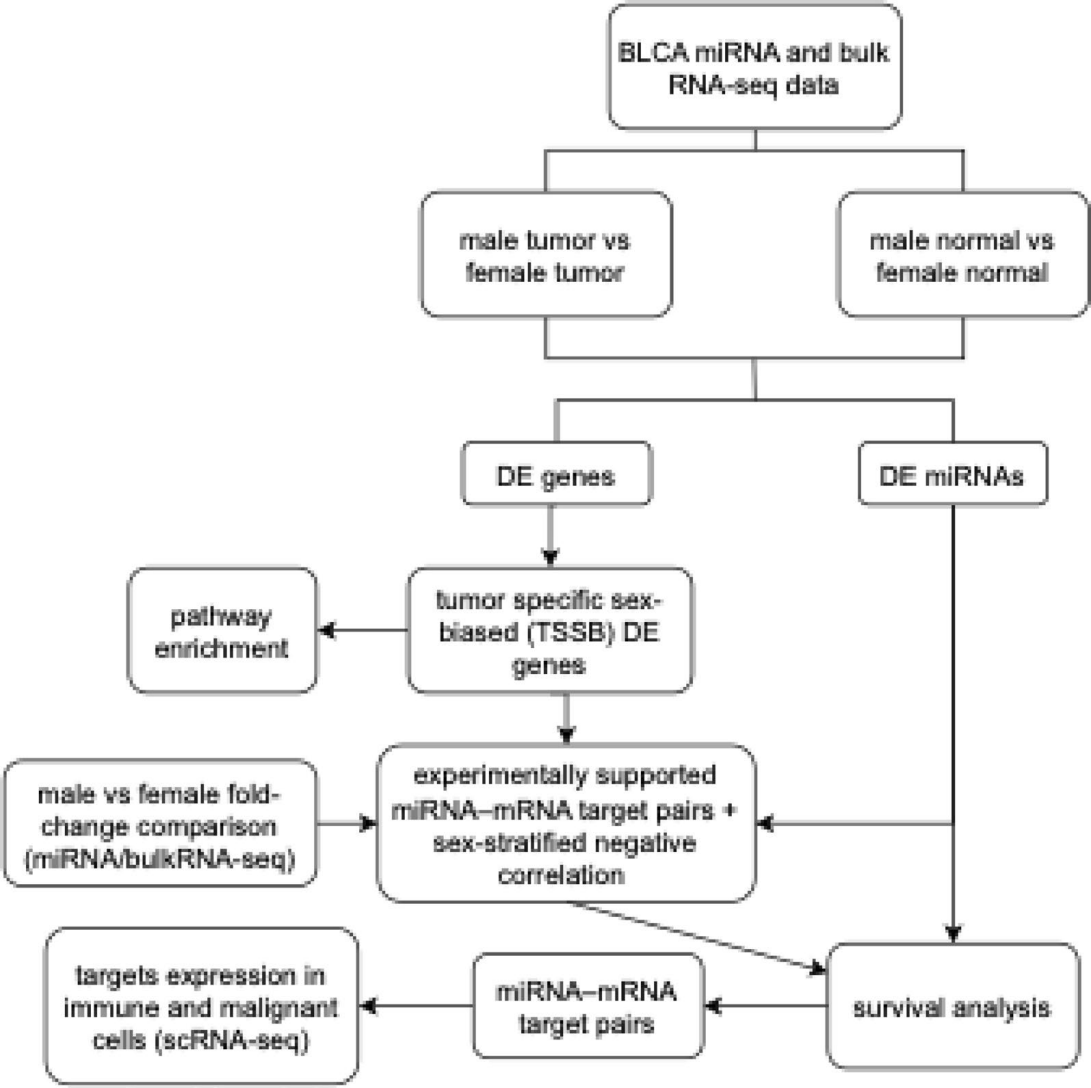
Overview of the integrative pipeline for identifying sex-biased miRNA–mRNA regulatory mechanisms in bladder urothelial carcinoma (BLCA). Differential expression (DE) analyses were performed on TCGA miRNA-seq and bulk RNA-seq data comparing male versus female tumors and normal samples. Tumor-specific sex-biased (TSSB) genes were defined from the tumor comparison (including genes with discordant tumor–normal directions) before being subjected to pathway enrichment. Experimentally supported miRNA–mRNA interactions (miRTarBase) were intersected with TSSB genes and evaluated using sex-stratified Pearson correlations; negatively correlated pairs were retained. Pairs were then classified by sex-bias direction via log2 fold change (log2FC); discordant pairs showed opposite directions (male-up miRNA with female-up mRNA, or vice versa). Kaplan–Meier/Cox survival analyses and scRNA-seq–based cell-type–specific expression were used to prioritize functional miRNA-target pairs with potential clinical relevance

We hypothesize that sex disparities in bladder cancer arise from regulatory programs best captured by integrating tumor-level transcriptional differences with post-transcriptional control and cell-type context. Accordingly, we conducted an integrative multi-omics analysis (Fig. 1), obtaining bulk RNA-seq, miRNA-seq, and clinical data for bladder urothelial carcinoma (BLCA) from The Cancer Genome Atlas (TCGA) via the Genomic Data Commons (GDC), and scRNA-seq data from the Genome Sequence Archive (GSA), with additional public resources (e.g., GEO) where applicable. Bulk RNA-seq quantifies sex-biased expression and defines tumor-specific sex-biased (TSSB) genes at the tumor level; miRNA-seq provides regulatory directionality by intersecting experimentally supported miRNA–mRNA pairs with sex-stratified negative correlations; and scRNA-seq resolves the cell-type context of these signals across epithelial, stromal, and immune compartments.

Building on sex-differential expression in both mRNAs and miRNAs, we prioritized functional axes using correlation-based target inference, evaluated clinical relevance with sex-stratified Kaplan–Meier survival analyses, and mapped TSSB genes across major tumor-infiltrating cell types to situate these programs within the tumor microenvironment. Together, this integrative approach uncovers sex-specific regulatory mechanisms in BLCA, identifies key sex-biased genes and miRNAs and their putative miRNA–mRNA interactions, links them to survival and cellular distribution, and nominates biomarkers and regulatory targets for sex-informed precision oncology.

## Materials and methods

### Data collection and preprocessing

RNA-seq, miRNA-seq, and clinical data for BLCA were downloaded from the Genomic Data Commons (GDC) (Data Release 42.0) using GDCquery and GDCdownload functions from the TCGAbiolinks R package (version 2.30.3) [24]. Clinical information, including sex, age at diagnosis, tumor stage, tumor grade, and survival status, was retrieved from the GDC portal. Samples with missing tumor grade or ambiguous stage information were excluded. In total, 293 male and 106 female tumor samples, and 10 male and 9 female adjacent normal samples were included for downstream analyses. The demographic and clinical characteristics of all included patients are summarized in Supplementary Table 1. Single-cell RNA-seq data were obtained from the Genome Sequence Archive (GSA-Human ID: HRA000212), comprising 4 male and 2 female BLCA tumor samples. Protein-coding genes and mature miRNAs were selected for downstream analyses. Raw sequencing reads were processed using standardized Nextflow RNA-seq and miRNA-seq analysis pipelines [25, 26] to generate raw count expression matrices. Lowly expressed genes and miRNAs were filtered out to improve statistical robustness. Principal component analysis (PCA) was performed using DESeq2 (version 1.42.0) [27] to evaluate global expression patterns and assess potential batch effects. No significant batch effects were observed in either the mRNA or miRNA datasets (Supplementary Fig. S1A-B).

### Differential expression (DE) analysis

DE analysis was conducted using DESeq2 (v 1.42.0) [27]. Normalization was performed with DESeq2’s median-of-ratios method to account for library size and compositional differences across samples. For mRNA-seq data, clinical variables including age, tumor stage, and tumor grade were incorporated into the model to adjust for potential confounding. Model performance assessment indicated that inclusion of these covariates improved dispersion estimates and overall fit. For miRNA-seq data, models were constructed without clinical covariates, as their inclusion did not improve model fit and introduced additional variability. The Wald test was used for differential expression analysis between males and females, separately for tumor and normal samples. Significant DE genes and miRNAs were selected when adjusted *P* < 0.05 and absolute log2 fold change (|log2FC|) > 0.58. Multiple testing correction was performed using the Benjamini-Hochberg method. The R packages pheatmap (v1.0.12) [28], gplots (v3.2.0) [29] and VennDiagram (v1.7.3) [30] were utilized to generate volcano plots, bar plots and Venn diagrams for DE results.

### GO and pathway enrichment analysis

Gene Ontology (GO) and the Kyoto Encyclopedia of Genes and Genomes (KEGG) pathway analyses were performed using clusterProfiler (v4.6.2) [31], with significance defined as an adjusted *P* < 0.05 and a minimum gene count ≥ 2. The top ten GO terms, KEGG pathways, and selected cancer-related pathways were visualized.

### Target prediction and correlation analysis

Experimentally validated miRNA–mRNA interactions were obtained from the miRTarBase (release 10.0) [32]. We retained only interactions with direct experimental evidence and excluded prediction-only pairs. Analyses were restricted to DE protein-coding genes and DE miRNAs identified in tumor samples. For correlation analyses, counts were normalized using DESeq2 variance-stabilizing transformation, and sex-stratified Pearson correlations were computed across tumor samples for each validated pair. Pairs were retained with significant negative correlations (Pearson *r* < 0; *P* < 0.05) in at least one sex. We annotated the direction of sex bias for each retained pair based on the sign of the male–female log2 fold-change, and reported the Pearson correlation coefficient and P value for each sex. Cytoscape (v3.2.0) [33] was used to construct and visualize the miRNA–mRNA regulatory network.

### Survival analysis

R packages survival (v3.8) [34], survminer (v0.5.0) [35], survivalROC (v1.0.3.1) [36], and coxphf (v1.13.4) [37] were used for survival analyses and the calculation of Cox proportional hazards ratios in BLCA tumor samples. Overall survival (OS) was defined as the time from diagnosis to death or last follow-up, with survival status coded as 1 (deceased) or 0 (censored). Patients were stratified by sex and dichotomized into “high” and “low” expression groups based on the median value for each feature. Kaplan–Meier (KM) curves with log-rank tests and univariate Cox proportional hazards models were used to assess survival differences between groups.

### Single cell RNA-seq data analysis

Raw sequencing data were demultiplexed and converted to FASTQ format using bcl2fastq v2.20. Cell Ranger v9.0.1 (10X Genomics) was used for barcode identification, read alignment, and unique molecular identifiers (UMIs) quantification with default parameters. Raw reads were aligned to the human reference genome (GRCh38), and gene expression counts were collapsed based on UMIs , resulting in a digital gene expression matrix with genes as rows and cell barcodes as columns. Low-quality cells and doublets were excluded based on the following thresholds: mitochondrial gene expression > 10%, total UMI count < 1000, and total detected features < 300 or > 6000. Each sample was log-normalized and integrated across batches using Harmony [38] to correct for batch effects, resulting in effective sample mixing and the removal of technical artifacts (Supplementary Fig. S3B). To obtain two-dimensional projections of cellular heterogeneity, principal component analysis (PCA) was performed on the integrated, normalized gene-barcode matrix using the top 5000 highly variable genes, selected based on mean expression and dispersion using the Seurat package (v5.3.0) [39, 40] in R (v4.2.0). Uniform manifold approximation and projection (UMAP) was then applied to visualize cellular heterogeneity in two dimensions. Unsupervised graph-based clustering was conducted to group cells, and cluster-specific marker genes from a previously published study were used to annotate cell populations [41].

### Code Availability

All code necessary to reproduce the analyses and figures of this manuscript is available on GitHub at https://github.com/CedarsDSN/BLCA_miRNA_mRNA_sex_diff.git.

## Results

### TCGA BLCA MiRNA DE analysis

**Fig. 2:**
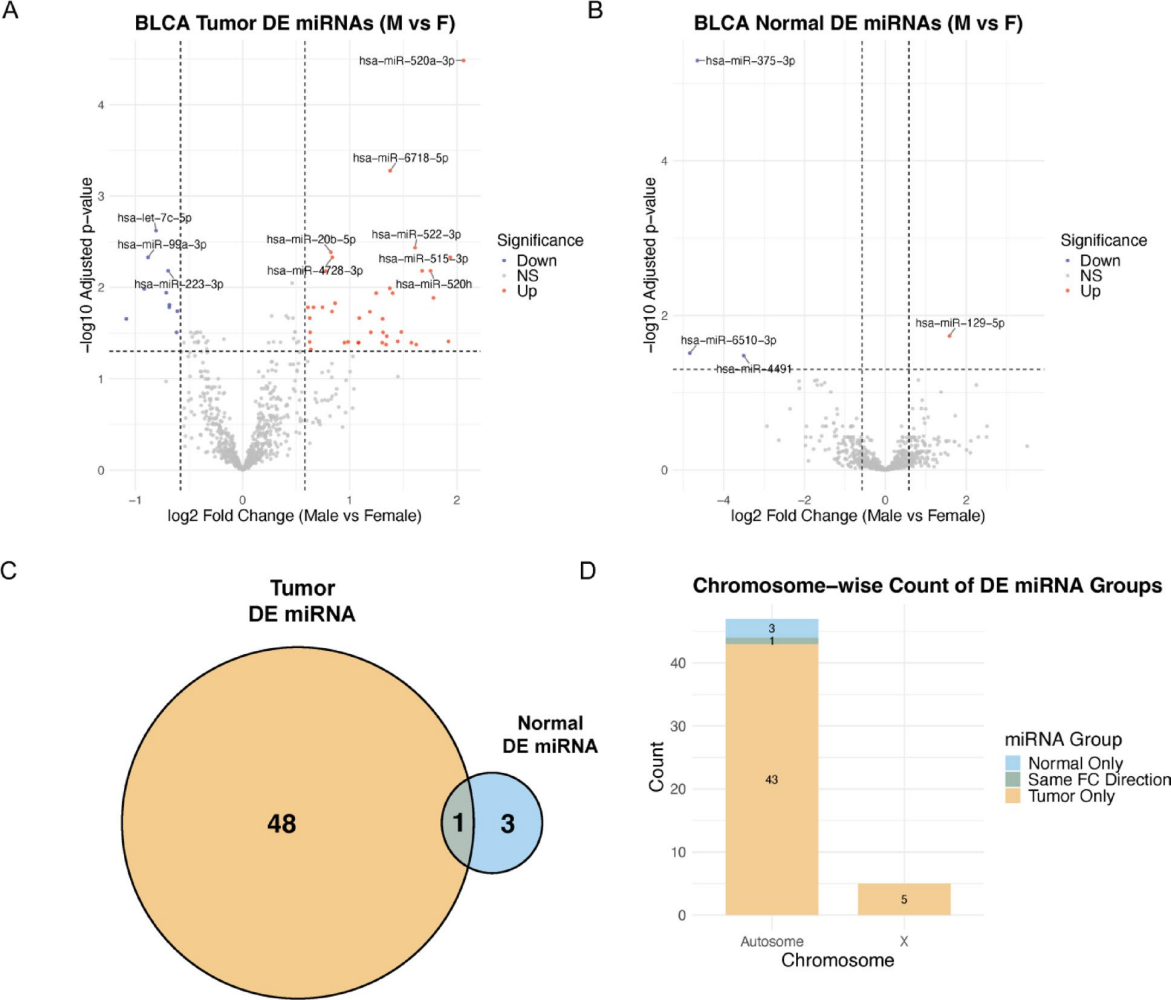
Sex-biased miRNA expression in BLCA tumors and normal samples. (**A–B**) Volcano plots comparing male vs. female in tumors (**A**) and normal bladder tissue (**B**). Significance was defined as adjusted *P *< 0.05 and |log₂FC| > 0.58; significantly upregulated (red) and downregulated (blue) miRNAs are highlighted. The top 10 significant miRNAs are labeled in (**A**), and all significant miRNAs are labeled in (B). Fewer DE miRNAs were observed in normal tissue than tumors. (**C**) Venn diagram showing the overlap of DE miRNAs between tumor and normal comparisons. (**D**) Chromosomal distribution of DE miRNAs for tumor and normal comparisons; most tumor-specific sex-biased miRNAs map to autosomes

After filtering out lowly expressed miRNAs, differential expression (DE) analysis of 803 miRNAs in BLCA tumor samples from 293 males and 106 females identified 49 significantly DE miRNAs (adjusted *P* < 0.05 and | log₂ fold change| > 0.58) (Supplementary Table 2). Specifically, 39 miRNAs were upregulated in males and ten in females (Fig. 2A, Supplementary Table 2). In contrast, analysis of adjacent normal bladder samples from 10 males and 9 females identified four DE miRNAs with the same significance threshold with three miRNAs upregulated in males and one in females (Fig. 2B). Notably, hsa-miR-129-5p was the only miRNA consistently differentially expressed in both tumor and normal samples and all other DE miRNAs were either tumor-specific or normal-specific sex-biased (Fig. 2C). Chromosomal distribution analysis showed that five tumor-specific DE miRNAs were located on the X chromosome and the remaining DE miRNAs were distributed across autosomes (Fig. 2D, Supplementary Table 2). The female-upregulated miRNA hsa-let-7c-5p acts as a tumor suppressor at the molecular level; however, clinically, its elevated expression has been previously linked to disease progression in high-grade bladder cancer [42]. The male-upregulated miRNA, hsa-miR-1270 has been shown particularly associated with apoptosis resistance and enhanced tumor cell survival [43, 44]. Another male-upregulated miRNA, hsa-miR-4728-5p, located within the HER2 locus, has been associated with increased proliferation and migration in cancer [45]. Excluding the single miRNA shared between tumor and normal tissues, the remaining 48 tumor-specific sex-biased (TSSB) miRNAs were selected for further analysis as potential sex-dependent regulatory elements involved in bladder cancer pathogenesis.

### TCGA BLCA mRNA DE analysis

**Fig. 3:**
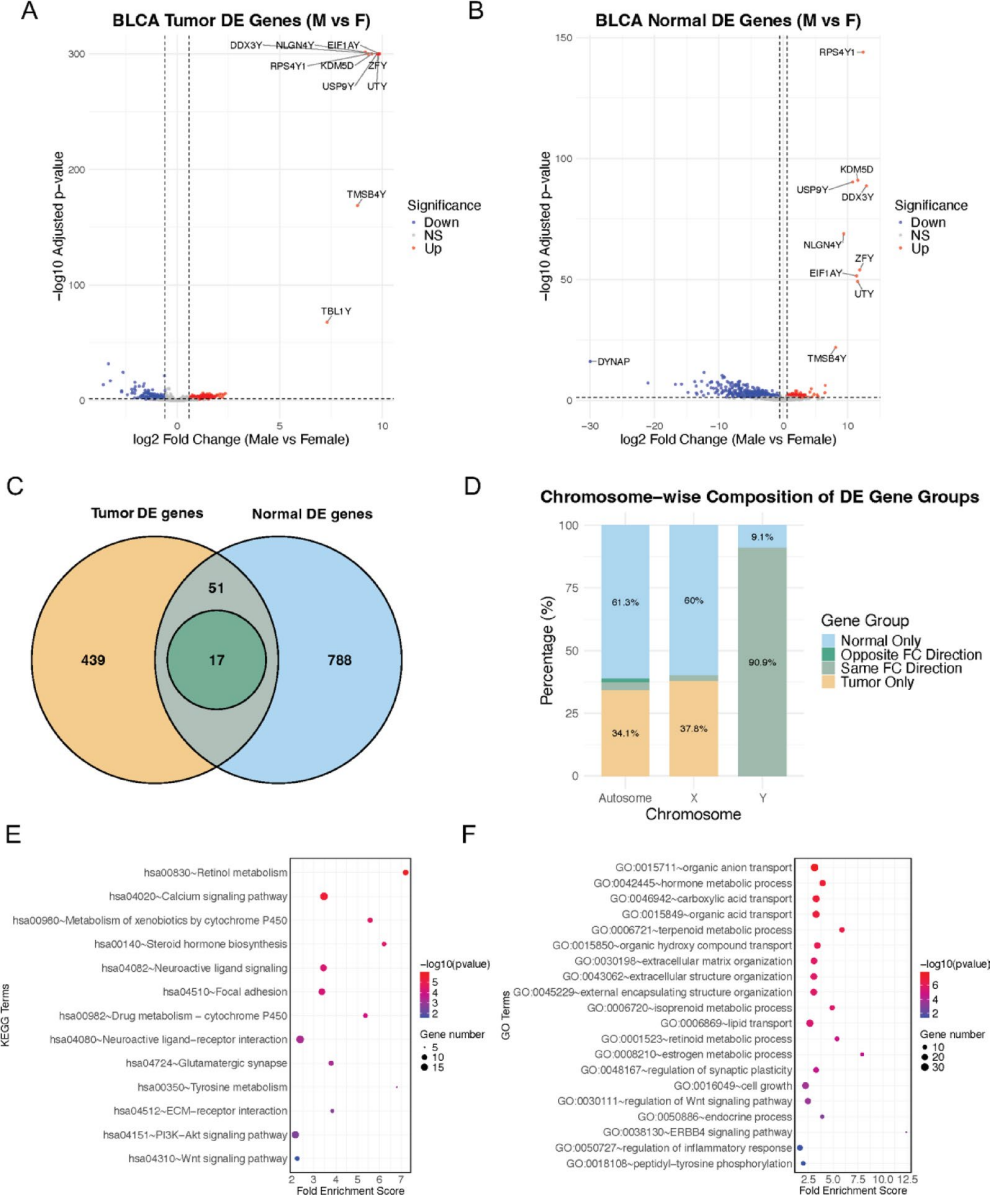
Sex-biased gene expression and pathway enrichment in BLCA tumor and normal tissue. Volcano plots of DE genes between male and female samples in BLCA tumors (**A**) and normal bladder tissues (**B**). Significantly upregulated (red) and downregulated (blue) genes are highlighted, with top significant genes labeled. (**C**) Venn diagram showing the overlap of DE genes between tumor and normal comparisons. (**D**) Chromosomal distribution of DE genes by tissue specificity. Overlapped DE genes with same fold change direction are located on the Y chromosome and tumor-specific or normal-specific DE genes are mostly located on autosomes and X chromosome. (E–F) Pathway enrichment analysis of TSSB genes using KEGG (**E**) and GO terms (**F**) demonstrates enrichment in immune signaling, extracellular matrix organization, and hormone-related pathways

Gene expression data for 19,409 protein-coding genes from BLCA were obtained from TCGA, and genes with low expression were filtered out prior to analysis. To investigate sex-specific differences in gene expression, we conducted differential expression analyses independently for tumor and normal samples, comparing males versus females within each sample type. For the tumor-specific comparison, tumor stage, tumor grade, and patient age were included as covariates in the model. This analysis identified 507 differentially expressed genes (DEGs) at thresholds of adjusted *P* < 0.05 and |log₂ fold change| > 0.58 (Fig. 3A). For normal samples, patient age was considered as a covariate, yielding 856 DEGs under the same significance thresholds (Fig. 3B). Comparisons between the two sets of DEGs identified 439 tumor-specific DEGs, 788 normal-specific DEGs, and 68 overlapping DEGs (Fig. 3C). Among these overlapping DEGs, 51 genes showed the same fold change directions, while 17 genes demonstrated opposite directions. Chromosomal distribution analysis revealed that 90.9% of overlapping DEGs with the same fold change direction were localized to the Y chromosome, suggesting expected sex-biased gene expression. In contrast, the tumor-specific and normal-specific DEGs were predominantly located on autosomes and the X chromosome, and all 17 genes with opposing fold change directions were autosomal (Fig. 3D). We designated the 439 tumor-specific DEGs and the 17 overlapping, direction-reversed DEGs as TSSB genes (456 total; Supplementary Table 2). Specifically, genes showing discordant sex-bias directions between tumor and normal tissues were included, as they represent a reversal of the sex differences observed in normal tissue. These TSSB genes are of particular interest, as they likely capture tumor-intrinsic sex differences in gene regulation and may contribute to distinct biological mechanisms and disease progression in male and female BLCA patients. These genes were selected for subsequent downstream analyses.

Enrichment analysis of Gene Ontology (GO) biological process and KEGG pathways showed that the TSSB genes were significantly enriched in diverse functional categories, including transport and metabolic processes, developmental pathways, extracellular matrix organization, structural remodeling, signal transduction, cell adhesion and migration, and immune and inflammatory responses (Fig. 3E-F, Supplementary Table 3). Notably, multiple cancer-related pathways were prominently represented, such as the regulation of Wnt signaling, ERBB4 signaling, inflammatory response regulation, and mechanisms associated with cell proliferation and growth. Hormone-related metabolic processes were also significantly enriched, including estrogen metabolism, hormone biosynthesis, and retinoid metabolism, suggesting a potential connection between sex hormones and sex-specific tumor biology in bladder cancer.

### MiRNA target prediction and regulatory network

**Fig. 4:**
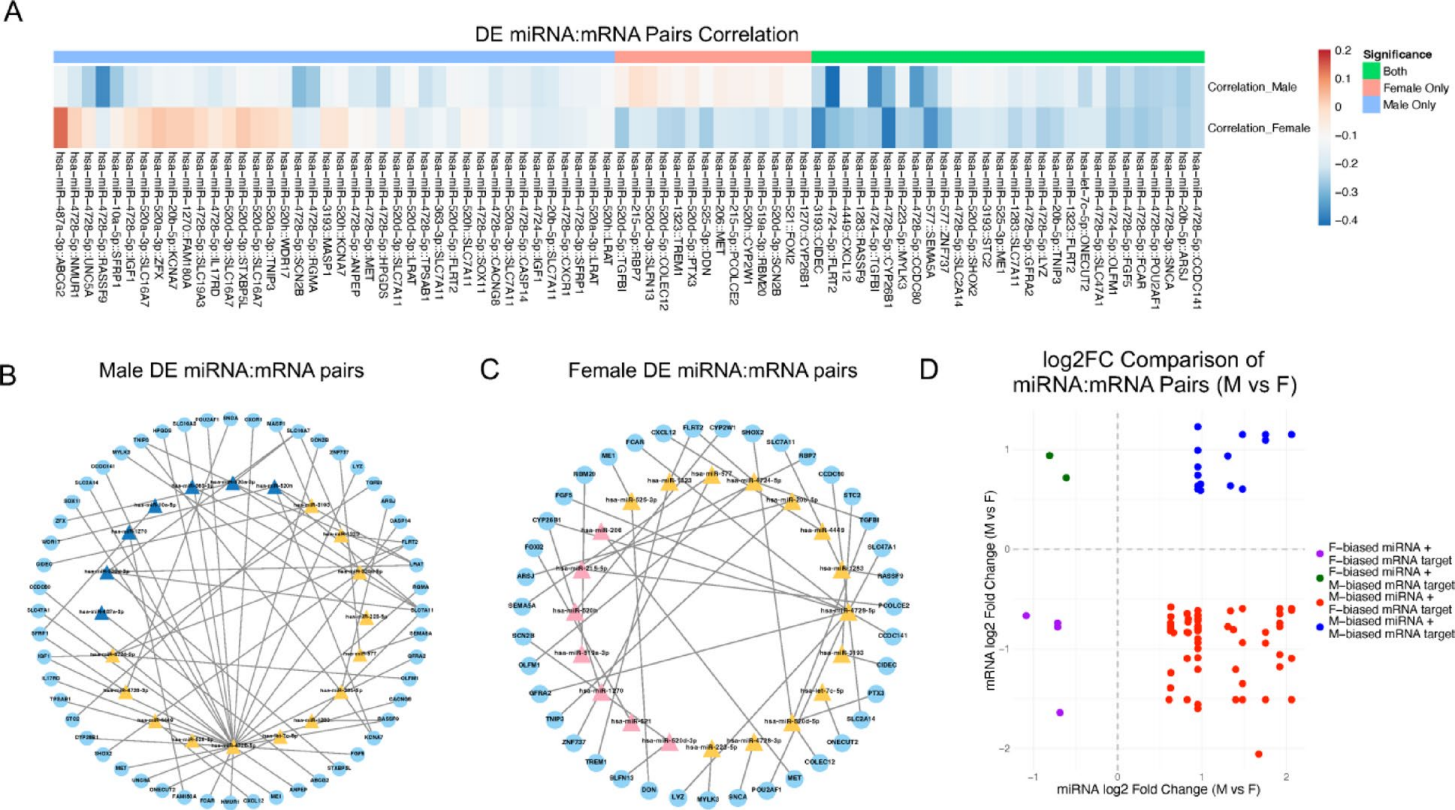
Sex-specific correlation, regulatory networks, and expression patterns of TSSB miRNA–mRNA pairs in BLCA tumors. (**A**) Heatmap showing Pearson correlation coefficients for experimentally supported TSSB miRNA–mRNA pairs in male and female BLCA tumors. Only pairs with significant negative correlations in either or both sexes are shown. (**B–C**) Regulatory networks of negatively correlated TSSB miRNA–mRNA pairs in male (**B**) and female (**C**) tumors. miRNAs (triangles) and target mRNAs (circles) are connected by edges representing validated negative correlations. miRNAs are colored blue (male-specific), pink (female-specific), or yellow (shared between sexes). (**D**) Scatter plot comparing log2 fold changes of miRNAs and their target mRNAs between male and female tumors. Each point represents a TSSB miRNA–mRNA pair, color-coded by regulatory category (e.g., male-biased miRNA with female-biased mRNA target)

Following the identification of sex-biased expression patterns in mRNAs and miRNAs in BLCA, we constructed an integrated regulatory network to examine coordinated interactions. Our analysis focused on the 48 TSSB miRNAs and 456 TSSB genes. To identify functional miRNA–mRNA regulatory relationships, we used the miRTarBase database, which contains experimentally validated miRNA–mRNA interactions [32]. Candidate pairs were restricted to TSSB miRNAs and TSSB genes that matched validated interactions in miRTarBase. To further refine this set, we calculated Pearson correlation coefficients between each TSSB miRNA and its validated TSSB mRNA target in tumor samples, retaining only pairs with significant negative correlations, a hallmark of canonical miRNA-mediated gene repression. This analysis yielded 82 negatively correlated, experimentally supported TSSB miRNA–mRNA pairs (Fig. 4A-C, Supplementary Table 4). Notably, several TSSB miRNA–mRNA pairs demonstrated sex-biased correlation patterns: some pairs showed significant negative correlation exclusively in either male or female samples, while others were significant in both sexes. These findings indicate the presence of sex-dependent miRNA-mediated repression of gene expression in bladder cancer.

We further categorized the 82 miRNA-mRNA target pairs based on the direction of differential expression in tumors, as determined by log2 fold change. The pairs were grouped into four categories: (1) female-biased miRNAs with female-biased mRNA targets, (2) female-biased miRNAs with male-biased mRNA targets, (3) male-biased miRNAs with female-biased mRNA targets, and (4) male-biased miRNAs with male-biased mRNA targets. The pairs of primary interest were in the discordant groups, female-biased miRNAs with male-biased mRNA targets, and vice versa, as these pairs likely represent sex-specific regulatory interactions. Among the discordant groups, we identified two pairs of female-biased miRNAs with male-biased mRNAs, and 61 pairs of male-biased miRNAs with female-biased mRNAs. These discordant interactions likely suggest high-confidence candidates of sex-specific post-transcriptional regulation in bladder cancer (Fig. 4D). Notably, hsa-miR-4728-5p, highly expressed in male tumors, accounted for 26 of the 61 discordant pairs, suggesting a prominent regulatory role in male-specific gene repression. Several of its TSSB targets, including IGF1, SFRP1, and MET, are well-known cancer-related genes involved in growth factor signaling, Wnt pathway regulation, and tumor progression. Additionally, several discordant miRNA–mRNA pairs have been previously identified in cancer-related studies, such as miR-520d-5p–SLC7A11, associated with ferroptosis resistance in oral squamous cell carcinoma [46].

### Survival analysis

**Fig. 5:**
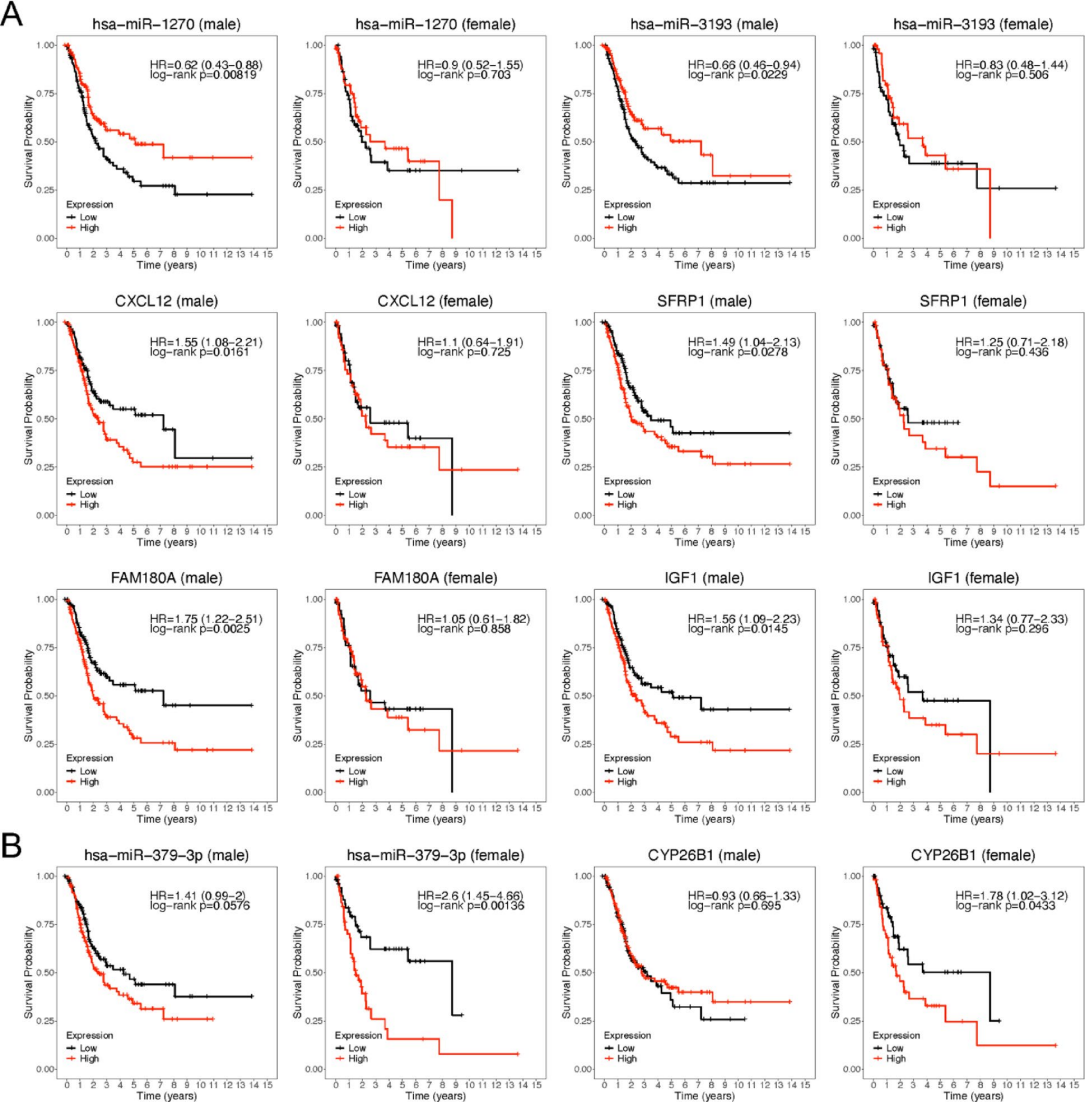
Sex-specific survival analysis of TSSB miRNAs and genes in BLCA patients. Kaplan–Meier survival curves for TSSB miRNAs and selected TSSB targeted genes significantly associated with overall survival in (**A**) male (*n* = 293) or (**B**) female (*n* = 106) BLCA patients. Patients were stratified into high (red) and low (black) expression groups based on median expression. Log-rank p-values and univariate Cox proportional hazard ratios (HRs, 95% CI) are shown. Statistical significance was defined as *P* < 0.05

Kaplan–Meier survival analysis was performed on the 48 TSSB miRNAs and 456 TSSB genes. We defined a gene or miRNA as sex-favored or -unfavored based on whether higher expression was associated with better or worse survival in males or females, respectively. Several TSSB miRNAs demonstrated significant sex-specific associations with overall survival. For example, hsa-miR-1270 and hsa-miR-3193 were significantly associated with improved survival in male patients only, suggesting they function as male-favored miRNAs (Fig. 5A). In contrast, hsa-miR-379-3p was significant only in females, with higher expression associated with poorer survival, indicating a female-unfavored miRNA (Fig. 5B). High expression of hsa-let-7c-5p was significantly associated with poor survival in both sexes (Supplementary Fig. S2A).

Among the 456 TSSB genes, 72 were significantly associated with survival in males only, 17 in females only, and 5 in both sexes (Supplementary Table 5). Of the male-specific survival-associated genes, 9 were predicted targets of TSSB miRNAs. In contrast, only one predicted target was uniquely associated with survival in females, while three predicted target genes were significant in both sexes (Fig. 5A-B; Supplementary Fig. S2A-B). Notably, all 13 target genes were TSSB genes and were consistently upregulated in female tumors. The interactions between these miRNAs and their survival-associated TSSB targets were supported by miRTarBase and miRWalk (Supplementary Table 6). The pairs hsa-miR-1270–CYP26B1 and hsa-miR-1270–FAM180A involved differentially expressed survival-associated miRNAs and TSSB genes, both significant in males, highlighting them as strong candidates for sex-specific regulatory mechanisms in BLCA.

### Single cell RNA-seq analysis

**Fig. 6:**
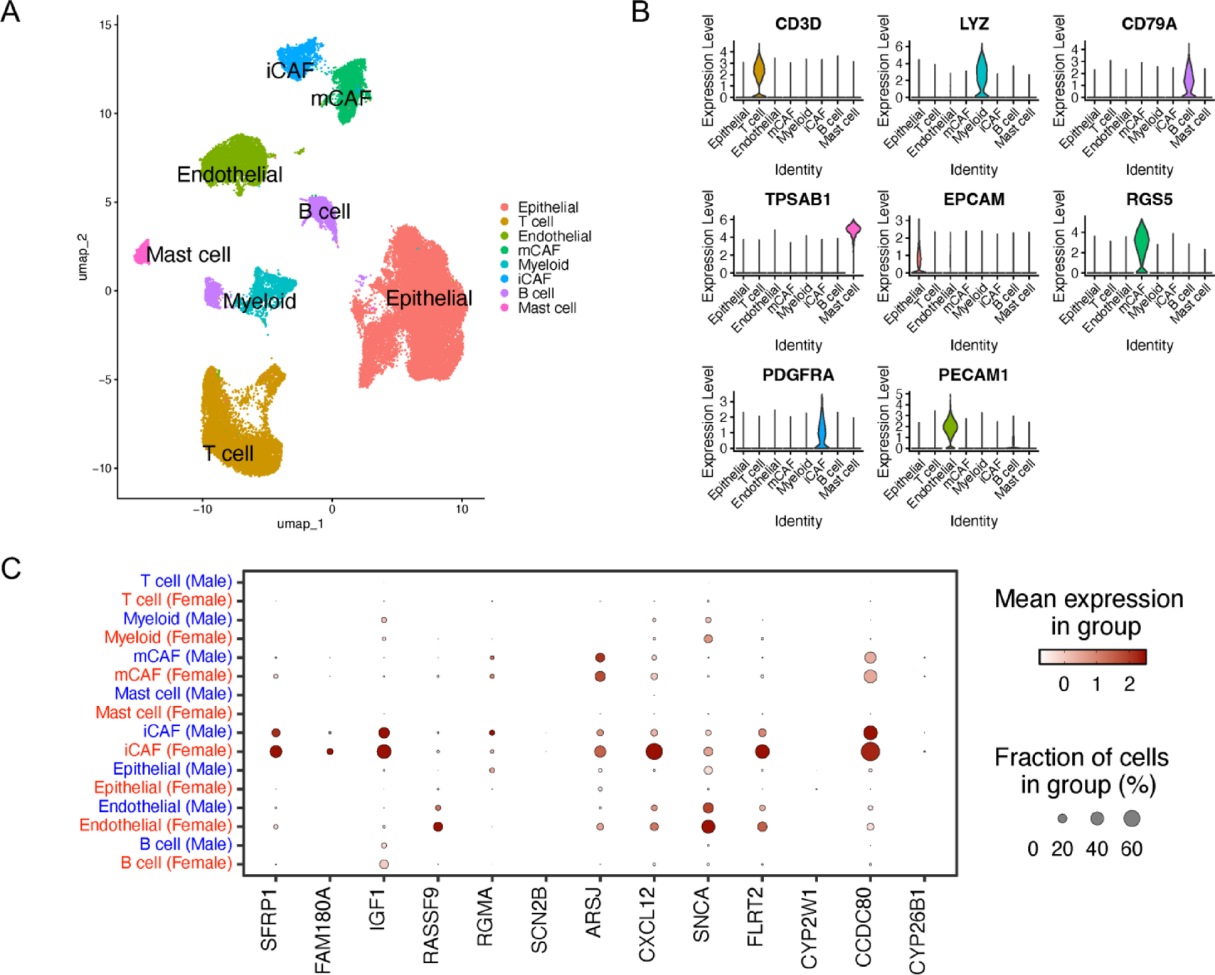
Cell type annotation and sex-specific expression patterns in single-cell BLCA tumors. (**A**) UMAP plot showing major cell populations identified in BLCA tumors, including epithelial cells, endothelial cells, myeloid cells, mast cells, T cells, B cells, inflammatory cancer-associated fibroblasts (iCAFs), and myofibroblastic CAFs (mCAFs). (**B**) Violin plots of canonical marker genes used to define each cell type. (**C**) Dot plot showing the expression of selected TSSB genes across cell types, split by sex. Dot size reflects the percentage of cells expressing each gene within the group, and color indicates average expression level

To understand the cellular landscape underlying sex-biased gene expression in bladder cancer, we analyzed single-cell RNA-seq data from male and female tumor samples. We focused on the 13 TSSB target genes associated with survival outcomes to perform differential expression analysis across each cell type. Using canonical markers, we annotated eight cell populations (Fig. 6A-B): epithelial cells (EPCAM+), inflammatory cancer-associated fibroblasts (iCAFs; PDGFRA+), T cells (CD3D+), endothelial cells (CD31+), myo-cancer-associated fibroblasts (mCAFs; RGS5+), myeloid cells (LYZ+), B cells (CD79A+), and mast cells (TPSAB1+), consistent with a previous study [41]. The final dataset comprised 47,631 cells, with the distribution of cell counts across sex and lineage summarized in Supplementary Fig. S3A.

We identified consistent sex-biased expression across these cell types for the 13 TSSB genes using a significance threshold of adjusted *P* < 0.05 and |log2FC| > 0.58 (Supplementary Table 7). Widespread female-biased expression was observed in endothelial cells, B cells, iCAFs, and T cells, consistent with the discordant pattern of male-biased miRNAs paired with female-biased mRNAs (Fig. 6C). In B cells and iCAFs, CXCL12 and IGF1 were consistently downregulated in males, suggesting reduced chemokine and growth factor signaling in the male tumor microenvironment. Endothelial cells showed strong female-biased expression of genes including SFRP1, IGF1, RASSF9, and FLRT2, which are associated with angiogenesis, immune regulation, and Wnt pathway inhibition. In iCAFs, genes such as CXCL12, FAM180A, and FLRT2 showed higher expression in females, suggesting more active stromal signaling activity and extracellular matrix remodeling in female tumors. In contrast, epithelial cells demonstrated a mixed pattern of sex-biased gene expression. Male-upregulated targets included CCDC80, FLRT2, IGF1, CXCL12, SNCA and RGMA, many of which are associated with proliferation, metabolic regulation, and immune modulation, while female-upregulated genes such as RASSF9 and CYP2W1 are associated with cell growth regulation and metabolic processes. These results indicate that sex-biased gene expression in bladder cancer differs by cell type. Female tumors exhibited higher expression of several genes in stromal and immune cells, while epithelial cells showed a mix of male- and female-biased patterns, suggesting distinct regulatory programs between sexes.

## Discussion

Bladder cancer shows significant sex disparities in both incidence and outcomes: males develop BLCA roughly fourfold more often than females [47]. However, female patients more often present with more advanced disease and poorer survival [48]. To investigate the molecular basis of these clinical differences, we performed an integrative multi-omics analysis, incorporating bulk mRNA and miRNA expression, experimentally supported miRNA-mRNA interactions, sex-stratified survival analyses, and single-cell transcriptomics.

We identified 48 tumor-specific sex-biased (TSSB) miRNAs when comparing male and female tumors, with approximately 90% located on autosomes. This suggests that miRNA-mediated sex biases in BLCA are likely driven by epigenetic or hormonal regulation rather than sex-chromosome dosage. This pattern is expected given dosage compensation of the X chromosome and the limited protein-coding repertoire of the Y chromosome in somatic tissues. These observations are consistent with sex-linked cues—hormonal, epigenetic, and miRNA-mediated regulation—acting primarily on autosomal programs, and with prior work showing that sex-biased expression across human tissues is largely autosomal [49, 50]. Four TSSB miRNAs showed sex-specific associations with overall survival. Notably, hsa-miR-1270 and hsa-miR-3193 were upregulated in male tumors and associated with improved survival in males, suggesting male-favored, potentially tumor-suppressive roles. Although hsa-miR-1270 has been shown to promote cisplatin resistance by inhibiting apoptosis and to enhance tumor cell survival through the Cdr1as/miR-1270/APAF1 axis [43], and is associated with poor prognosis and metastasis in osteosarcoma and hepatocellular carcinoma [44], our findings suggest protective association in male BLCA. This effect may reflect androgen-mediated modulation of key oncogenic targets, possibly involving WT1-related pathways similar to those described in glioblastoma [51]. Similarly, hsa-miR-3193 has been reported as a favorable prognostic marker in uterine corpus endometrial carcinoma [52], consistent with our findings of its association with improved survival in male BLCA. In contrast, hsa-miR-379-3p was upregulated in female tumors and associated with poorer survival specifically in females. While the 5p arm of miR-379 has been well characterized as a tumor suppressor through targeting UBE2E3 in bladder and other cancers, the 3p arm remains understudied and may exert context-dependent or even opposing effects [53, 54]. Finally, elevated expression of hsa-let-7c-5p was associated with poor survival outcomes in both sexes. Multiple studies have shown that hsa-let-7c-5p is downregulated in early-stage BLCA and functions as a tumor suppressor via the PI3K/Akt/FoxO pathways, while higher urinary or tissue levels are associated with increased progression risk in non-muscle-invasive and high-grade tumors [42, 55, 56]. Its adverse prognostic association may reflect compensatory upregulation in aggressive subtypes, potentially contributing to therapy resistance or epithelial-to-mesenchymal transition [22].

To understand the downstream effects of TSSB miRNA regulation, we identified 456 TSSB genes by comparing male and female tumor samples while controlling for normal tissue differences. Over 95% of TSSB genes were located on autosomes, supporting previous findings that sex differences in cancer are primarily driven by regulatory mechanisms rather than direct effects of sex chromosome dosage [57–59]. Pathway enrichment analysis showed that TSSB genes were significantly enriched in tumor progression-related pathways, including extracellular matrix (ECM) remodeling, Wnt signaling, hormone metabolism, and immune regulation. The enrichment of the Wnt signaling pathway is consistent with our previous study, in which female-specific hub genes were significantly associated with Wnt signaling-related processes [60]. Notably, the Wnt inhibitor SFRP1, frequently silenced by methylation in BLCA, showed relatively higher expression in female tumors in our study. This indicates enhanced regulatory control of Wnt signaling in females, potentially contributing to sex-specific differences in tumor progression. We also identified the estrogen metabolism pathway as significantly enriched with TSSB genes. Estrogen metabolism has been shown to regulate multiple pathways involved in tumor progression and immune regulation, including Wnt signaling and chemokine expression. A previous study has shown that estrogen receptor signaling can upregulate the chemokine CXCL12 and its receptor CXCR4 in lung adenocarcinoma in a sex-dependent pattern [61]. In our study, CXCL12 was identified as a female-biased gene in immune and stromal cell populations, indicating that estrogen could be driving higher CXCL12 expression in the female tumor microenvironment. CXCL12 has been shown to recruit immune cells and also to facilitate metastasis via chemotactic gradients [62, 63] and may contribute to sex-specific patterns of immune infiltration or metastatic potential in BLCA. Together, these findings are consistent with an integrative model in which sex-hormone signaling influences ECM remodeling and modulates Wnt/β-catenin activity, helping to shape the stroma-rich, immune-enriched microenvironment observed in female tumors.

By intersecting the TSSB miRNAs and TSSB genes, we identified 82 experimentally validated, negatively correlated miRNA–mRNA target pairs. Of these, 63 were discordant pairs in which male-biased miRNAs targeted female-biased genes or vice versa, highlighting post-transcriptional regulation as a potential mechanism driving tumor-intrinsic sex differences in bladder cancer. Among these, hsa-miR-4728-5p (HER2 intronic) targets multiple TSSB genes such as SFRP1, IGF1, and MET. SFRP1, a known Wnt antagonist often silenced in advanced bladder cancer, may be repressed by miR-4728-5p in males, potentially enhancing Wnt/β-catenin signaling and promoting tumor progression [64]. Similarly, repression of IGF1 and MET, both involved in growth factor signaling and tumor progression, may contribute to male-specific oncogenic pathways and serve as potential therapeutic targets via IGF-1R or c-Met inhibition [65, 66]. Prior studies have characterized regulatory axes involving miRNAs identified in our analysis. For example, in OSCC, circFNDC3B enhances malignancy by sponging miR-520d-5p, leading to upregulation of SLC7A11, which inhibits ferroptosis [46]. Collectively, these discordant interactions suggest that male-biased miRNAs are associated with lower expression of oncogenic programs, such as growth factor signaling, that are otherwise more active in female tumors.

We hypothesize that these discordant miRNA–mRNA patterns reflect sex-dependent post-transcriptional regulation involving hormonal signaling, epigenetic states, and tumor microenvironmental factors. First, sex-hormone receptor signaling can modulate transcription of miRNA loci and/or their targets; for example, androgens may upregulate male-biased miRNAs (e.g., miR-1270) to repress downstream targets, while the absence of this hormonal drive in females leads to target derepression [51, 67]. Second, epigenetic regulation, such as differential DNA methylation, has been shown to silence genes like SFRP1 and SNCA in bladder cancer and may similarly modulate the promoters of sex-biased miRNAs [64, 68]. Third, the tumor microenvironment likely contributes to these bulk tissue observations, consistent with our single-cell findings in which female-biased targets like CXCL12 were predominantly localized to stromal and immune cells, contrasting with the epithelial expression of their regulatory miRNAs [41, 69]. While these explanations represent mechanistic hypotheses supported by prior studies, further functional studies are necessary to causally link these upstream drivers to the specific miRNA–mRNA interactions identified in our analysis.

To evaluate clinical relevance, we identified 13 targeted TSSB genes that showed both sex-biased expression and sex-specific associations with survival. Nine genes showed male-only survival significance, including SFRP1, FAM180A, IGF1, RASSF9, RGMA, SCN2B, ARSJ, CXCL12 and SNCA; one gene, CYP26B1 showed female-only significance and three genes (CCDC80, FLRT2, CYP2W1) were prognostic in both sexes. Notably, all these 13 genes showed higher gene expression in female tumors, and their higher expression was uniformly associated with poorer survival, indicating predominant oncogenic or risk-promoting functions in disease progression. This pattern demonstrates functional coherence between the regulatory data and clinical outcomes: the absence of repressive male-biased miRNAs in females correlates with upregulation of high-risk genes and poorer survival. This suggests that female-biased upregulation of risk genes may underlie the observed clinical aggressiveness in females [70, 71]. These 13 genes span diverse functional categories related to cancer pathogenesis. IGF1, encoding an insulin-like growth factor, drives tumor cell proliferation and survival through IGF1R activation and the downstream PI3K–Akt cascade—a pathway often dysregulated in urothelial carcinomas and linked to disease progression [72, 73]. Several genes act as tumor suppressors or adhesion modulators. RASSF9, a Ras-association family member, promotes proliferation via MEK/ERK; its female bias warrants investigation of estrogen-driven roles [74, 75]. RGMA curbs angiogenesis and cell migration via neogenin-mediated suppression of VEGF and FAK signaling, and its overexpression in oral squamous carcinoma cells markedly restrains proliferation, migration, and invasion [76]. SCN2B, a gene involved in neural and neuroendocrine processes, has been identified in cancer cell migration and neuroinvasion [77]. Other genes contribute to immune evasion or therapeutic resistance. SNCA, downregulated in BLCA via methylation, correlates with worse survival and immune infiltration at higher levels; its female bias may foster suppressive niches or clonal selection [68]. ARSJ, a lysosomal sulfatase, could alter ECM composition and cellular detox processes, while CYP26B1, which metabolizes retinoic acid, increasingly stands out as a promoter of tumor stemness and immune suppression in bladder cancer [67, 78, 79]. Collectively, these genes highlight the involvement of diverse biological pathways, including growth factor signaling, Wnt pathway inhibition, immune regulation, RA breakdown, and neuroendocrine adaptation, which contribute to sex-specific differences in BLCA progression.

Single-cell RNA-seq further revealed distinct expression patterns across cellular populations. For example, IGF1 showed male-biased expression in malignant epithelial cells, consistent with its role as a mitogenic driver modulated by androgens [66, 72]. Similarly, CXCL12, a chemokine with dual immune-recruiting and pro-metastatic functions, was higher in female fibroblasts, potentially driven by estrogen signaling [77, 78]. Prior studies, including single-cell RNA-seq analyses, have demonstrated that CXCL12—primarily expressed by inflammatory cancer-associated fibroblasts (iCAFs) in bladder cancer—promotes recruitment and accumulation of tumor-associated macrophages (TAMs) via its interaction with CXCR4 and other receptors such as CXCR3, DPP4, and ACKR3/CXCR7, thereby shaping immune infiltration in the tumor microenvironment. Notably, CXCL12 expression is positively correlated with TAM signature scores in the TCGA BLCA cohort and is associated with poor prognosis, while immunofluorescence studies confirm its production by iCAFs in tumor tissues, further supporting its role in modulating the tumor immune landscape and disease progression [41].

The miR-1270–CYP26B1/FAM180A axis exhibited sex-biased differential expression in both the miRNA and its target genes, all of which were associated with sex-specific survival outcomes. This exemplifies how sex-biased miRNAs may confer prognostic impact by repressing oncogenic targets. Both targeted genes showed higher expression in female tumors and were associated with poor male survival, suggesting that elevated miR-1270 in males may mitigate risk by suppressing these transcripts. This axis also underscores the potential for sex-specific therapeutics: for example, combining retinoid agonists with CYP26B1 inhibitors in female patients, or enhancing miR-1270 function in male tumors to curb angiogenesis and stromal activation [78, 80].

Our study highlights sex-specific molecular features in bladder cancer that have clinical relevance for prognosis and support the development of personalized treatment strategies. The identification of TSSB miRNAs and their TSSB mRNA targets, including several associated with survival, represents promising candidates for sex-specific prognostic biomarkers. For example, male-upregulated miRNAs such as hsa-miR-1270 and hsa-miR-3193, which are associated with improved survival in males, and discordant miRNA–mRNA pairs like miR-4728-5p–SFRP1/IGF1 may represent sex-dependent molecular signatures with therapeutic relevance. At the cellular level, single-cell RNA-seq showed that sex-biased gene expression spans epithelial, stromal, and immune cell populations, suggesting that differences in the tumor microenvironment between males and females may impact treatment response. These findings highlight the importance of considering sex as a biological factor in biomarker development, clinical trial design, and personalized therapy strategies for bladder cancer.

Limitations of our study include the unequal sample size between male and female patients in TCGA and the small number of female cases in the scRNA-seq cohort, which may constrain statistical power. Because TCGA smoking annotations are incomplete and molecular subtypes are expression-derived—and may mediate rather than confound—some residual confounding cannot be excluded; accordingly, we interpret sex effects alongside covariate-adjusted bulk analyses and concordant stromal/immune patterns in scRNA-seq. Although we prioritize experimentally supported miRNA–mRNA pairs, these regulatory relationships require functional validation. Nevertheless, our integrative approach, spanning transcriptome-wide differential expression analysis, target prediction, survival modeling, and cell-type resolution, offers a robust and novel perspective on sex-biased BLCA biology.

In conclusion, our findings demonstrate that sex differences in bladder cancer are largely driven by regulatory interactions on autosomal genes, particularly involving miRNAs and their downstream targets in key tumorigenic pathways. By revealing how these networks intersect with survival and cell-type–specific expression, our study provides a foundational map of sex-biased molecular vulnerabilities and paves the way for sex-informed therapeutic strategies and biomarker development in BLCA.

## Supplementary Information

Below is the link to the electronic supplementary material.

Supplementary Material 1: Supplementary Table 1 – Demographic table for TCGA patients

Supplementary Material 2: Supplementary Figure (docx)

Supplementary Material 3: Supplementary Table 2 – Differential expression results for miRNA and bulk RNA

Supplementary Material 4: Supplementary Table 3 – GO and KEGG pathway enrichment results for TSSB genes

Supplementary Material 5: Supplementary Table 4 – TSSB miRNA–mRNA candidate pairs

Supplementary Material 6: Supplementary Table 5 – Kaplan–Meier and Cox survival analysis results for TSSB miRNAs and mRNAs

Supplementary Material 7: Supplementary Table 6 – Survival-associated TSSB targets validated by miRTarBase and miRWalk

Supplementary Material 8: Supplementary Table 7 – Sex-biased differential expression of survival-associated TSSB targets across cell types

## Data Availability

All code necessary to reproduce the analyses in the figures of this manuscript is available on GitHub at https://github.com/CedarsDSN/BLCA_miRNA_mRNA_sex_diff.git.
