# Supplementary material for: Integrative microRNA and transcriptome analysis reveals sex-specific molecular divergence in human bladder cancer
Source: Biol Sex Differ. 2026 Jan 23;17:29. doi: 10.1186/s13293-026-00829-5 (PMC12911049; doi:10.1186/s13293-026-00829-5)
Supplement: Supplementary file 2 — Supplementary Material 2: Supplementary Figure (docx) [file 13293_2026_829_MOESM2_ESM.docx]

Supplementary Figures

Supplementary Figure 1

Supplementary Figure S1. Principal component analysis (PCA) of TCGA BLCA expression by sex. (A) miRNA-seq; (B) bulk mRNA RNA-seq. Left panels show tumor samples; right panels show normal samples. Points are individual samples colored by sex (female, red; male, teal).

Supplementary Figure 2

Supplementary Figure S2. Sex-stratified survival analyses for additional miRNAs and TSSB target genes in TCGA BLCA. (A) Kaplan–Meier (KM) curves for hsa-let-7c-5p shown separately in males and females. (B) KM curves for additional target genes (ARSJ, RGMA, RASSF9, SCN2B, SNCA, FLRT2, CCDC80, CYP2W1), each displayed in males and females.

Supplementary Figure 3

Supplementary Figure S3. scRNA-seq overview and integration check. (A) Post-QC cell counts by major lineage, stratified by sex. (B) Harmony-integrated UMAP colored by sex; male and female cells are well mixed across clusters.
